# Disparities in cardiovascular disease outcomes and economic burdens among minorities in southeastern Virginia

**DOI:** 10.1186/s12872-025-04771-z

**Published:** 2025-04-24

**Authors:** El Moudden Ismail, Amidi Asra, Sharaf Alddin Reem, Bittner Michael, Zhang Qi

**Affiliations:** Old Dominion University, Norfolk, VA USA

**Keywords:** Cardiovascular Diseases_1_, Heart disease Management_2_, Healthcare Disparities_3_, Economic Impact_4_, Minority Health_5_, Healthcare Utilization_6_, Healthcare Costs_7_, Social determinants of Health_8_

## Abstract

**Background:**

Cardiovascular diseases are the leading cause of mortality in the United States, presenting significant public health challenges and financial burdens, particularly in Southeastern Virginia, where African American and Hispanic (AA&H) populations are disproportionately affected.

**Methods:**

This retrospective observational study analyzed data from 30,855 hospital discharges of AA&H patients across Southeastern Virginia from 2016 to 2020, focusing on individuals aged 18 to 85 with cardiovascular diseases. Utilizing the Virginia Health Information database, we examined demographic information, clinical data, and healthcare utilization patterns through hypothesis tests and regression models to explore associations between these variables and the economic impacts of cardiovascular diseases.

**Results:**

Heart failure and shock (47.2% of discharges) and cardiac arrhythmia and conduction disorders (12.3%) were the most prevalent cardiovascular conditions. Female patients incurred significantly higher charges than males across conditions (7.1% higher in heart failure, *p* < 0.0001; 8.8% higher in chest pain, *p* < 0.01). Younger patients (< 65 years) faced 8.5% higher charges for cardiac arrhythmia with procedures (*p* < 0.0001) and 5.2% higher charges for circulatory disorders (*p* < 0.05). Year of discharge consistently predicted increasing costs (standardized coefficient 0.816 for acute myocardial infarction, *p* < 0.0001). The presence of fluid and electrolyte disorders was associated with significantly higher charges across conditions (standardized coefficient 0.042 for heart failure, *p* < 0.0001; 0.051 for acute myocardial infarction, *p* < 0.0001).

**Discussion:**

The findings highlight the complex interplay between demographic characteristics and healthcare costs among AA&H populations, underscoring the need for targeted interventions. The significant economic impact observed calls for culturally competent healthcare strategies that can mitigate high costs and improve health outcomes. However, the retrospective, administrative nature of the data limits establishing causality, with potential misclassification of some conditions.

**Conclusion:**

This study provides crucial insights into cardiovascular disease management’s demographic and economic dimensions among AA&H populations in Southeastern Virginia. By identifying key factors contributing to healthcare disparities, the research supports the development of tailored interventions aimed at reducing the burden of cardiovascular diseases, thereby improving overall health equity and reducing economic strains on the healthcare system.

## Introduction

Cardiovascular diseases (CVDs) continue to be the leading cause of mortality both globally and in the United States, exerting significant impacts on public health and healthcare economics [1]. According to the American Heart Association’s 2023 report, CVDs were responsible for nearly a million deaths in the United States in 2020 alone, confirming their status as the nation’s primary health threat [1, 2]. The economic burden of these diseases is huge particularly in the United States, where healthcare systems face substantial increased costs, including direct medical expenses and indirect costs such as lost productivity.

In Virginia, the CVDs scenario is particularly grim, with the state ranked sixth in heart disease-related deaths, exhibiting a mortality rate of 167.2 per 100,000 individuals [3, 4]. Southeastern Virginia, with its substantial African American and Hispanic (AA&H) populations, which accounts for ~ 40% of the population [5], is an acute focal point for these challenges [6, 7]. In Southeastern Virginia, about one third of the population are AA [8] which is higher than the statewide AA population of ~ 20% [9] Hispanic population are around 7% of Southeastern Virginia population compared to ~ 10% of statewide [10]. Despite the pressing need, detailed regional data has a significant void that could elucidate local healthcare delivery patterns and patient outcomes [11–13]. This gap is particularly evident in addressing disparities within minority populations, where studies have repeatedly shown higher readmission rates among African Americans and other minorities compared to their White counterparts—differences driven by socioeconomic, literacy, and comorbidity disparities [14–16].

The significance of understanding the social determinants affecting CVD disparities, such as access to healthcare, environmental factors, and social support structures, has been increasingly recognized [17]. However, current research lacks comprehensive insights into heart disease management within minority groups in Southeastern Virginia, a metropolitan area with Norfolk, Virginia Beach, Chesapeake, Newport News, Hampton, Portsmouth, Suffolk, and Williamsburg cities and counties and more than two million populations.

This study seeks to fill this critical gap and uncover factors linked to heightened CVDs prevalence among AA&H communities as well as investigate how these factors influence the costs and outcomes of CVD treatments by leveraging the Virginia Health Information (VHI) database, a comprehensive dataset that includes socio-demographic characteristics, clinical parameters, and healthcare utilization patterns.

Therefore, this study aims to estimate the prevalence and explore the demographic variability associated with CVD, assess the economic burden associated with CVD treatment, aiming to pinpoint how age, gender, and insurance type affect disease prevalence and treatment costs within predominantly minority communities. By examining these factors within the context of a primarily African American and Hispanic population, we provide insights into both disparities between demographic groups within these communities and the broader implications for minority health.

Furthermore, it investigates the role of comorbidities and healthcare usage in shaping patient outcomes. It offers a detailed evaluation that could guide the development of tailored healthcare strategies and policy measures designed to mitigate disparities and enhance CVD management across this diverse community.

## Materials and methods

This study utilized the VHI Patient Level Database, which contains all submitted, processed, and verified inpatient hospital discharges in the Commonwealth of Virginia. VHI has collected, analyzed, and distributed these public use files as valuable health care information tools since 1993. This extensive database includes detailed information on patient demographics, administrative records, clinical data, and financial details for each hospital discharge. This retrospective observational study was conducted using VHI data. It included AA&H patients aged 18 to 85 with CVDs covered by Medicare, Medicaid, self-pay, or other insurance types between 2016 and 2020. This study exclusively focused on AA&H populations in Southeastern Virginia, with no inclusion of other racial/ethnic groups. This population focus was intentional, as addressing cardiovascular health disparities in these specific minority communities in Hampton Roads was the primary aim of our research. Records were extracted based on the location of the hospitals they were admitted to, all of which were situated in Southeastern Virginia, including Norfolk, Virginia Beach, Chesapeake, Newport News, Hampton, Portsmouth, Suffolk, and Williamsburg. This regional focus allowed us to examine patterns specific to this metropolitan area with its significant AA&H population. This study also utilized the VHI Readmissions and Transfers Supplemental Data Set (RATs), which is linked to the Patient Level Database. To identify readmissions, we used VHI’s RATs dataset which defines readmissions as hospital stays that follow an earlier hospitalization by at least one day but within 90 days. Transfers, defined as hospital stays where the discharge date of the first admission is the same as the admission date of the second admission, were also analyzed.

The study population was defined by using Major Diagnostic Categories (MDCs) code 05 and Diagnosis-Related Groups (DRGs) Principal Diagnosis codes 280–316, which encompass patients with diseases and disorders of the circulatory system, as specified by the Centers for Medicare & Medicaid Services (CMS).

This study was determined to not involve human subjects research by the Eastern Virginia Medical School Institutional Review Board (IRB # 23-05-NH-0118) and therefore was deemed exempt from IRB review, including the use of VHI data. The need for informed consent was waived due to the retrospective nature of the study using de-identified data. Data was received by secure transfer and stored on password protected devices with access restricted to authorized research team members. In accordance with VHI data use requirements, all data were handled in a confidential manner and no attempts were made to identify, disclose, discuss, release, or provide access to information on specific individual patients. VHI has provided non-confidential patient level information used in this study which it has compiled in accordance with Virginia law but which it has no authority to independently verify. By using this data, the authors agree to assume all risks that may be associated with or arise from the use of inaccurate data. VHI cannot and does not represent that the use of VHI’s data was appropriate for this study or endorse or support any conclusions or inferences that may be drawn from the use of VHI’s data. Access to the VHI Patient Level Database and RATs was obtained through the M. Foscue Brock Institute for Community and Global Health at Macon & Joan Brock Virginia Health Sciences at Old Dominion University, which maintains a license for these data.

Data cleaning involved checking for inconsistent data. Upon thorough examination, we found that the VHI database provided complete data for all 30,855 hospital discharges included in our analysis. There were no missing values for any variables of interest, including total charge (USD) and all independent parameters (demographic, clinical, and administrative characteristics). This data completeness eliminated the need for imputation methods or other techniques to address missing values. All statistical analyses were conducted with EVMS-Research and Infrastructure Service Enterprise (RISE) using SAS version 9.4 (SAS Institute, Cary, NC) and R.

Descriptive statistics were utilized to summarize variables: quantitative variables including age, length of stay, pre and pos operative length of stay, and total charge were summarized by mean & Standard deviation (SD), or median & Interquartile Range (IqR) based on normality of the data. Categorical variables including gender, race, insurance, diagnosis, admission and source types, comorbidity, primary procedure, and patient city were summarized by frequencies and percentages [18].

Associations between categorical variables were assessed using the Chi-squared test or Fisher’s exact test, as appropriate [18, 19]. To address multiple comparisons and control the false positive rate, the Benjamini-Hochberg method was applied [20]. Correlation between quantitative variables was evaluated using Pearson’s or Spearman’s correlation tests, as appropriate [21].

To avoid multicollinearity, we removed the variable with the largest variance inflation factor (VIF) > 10 until all VIF values were below 10. Various regression models were implemented for normally distributed total charge, with robust regression methods such as quantile regression or generalized linear models (e.g., Poisson, negative binomial) employed for non-normally distributed total charge, incorporating the interaction of all possible factors as predictors of hospitalization cost and then testing the interaction’s *p*-value [18, 19, 22].

To compare the mean hospitalization costs between the two samples, a T-test for independent samples was performed. If the data distribution was skewed, the hospitalization cost data were transformed to achieve normality, or alternatively, the Wilcoxon rank-sum test was used.

For comparisons involving multiple groups, one-way ANOVA, Mood’s median test, or Kruskal-Wallis test was employed, depending on normality of the data [18, 23]. Statistical significance was determined by a *p*-value of < 0.05, categorized as follows: a *P* value ≤ 0.0001 is denoted as ‘a,’ 0.0001 ≤ *P* value < 0.01 as ‘b,’ and 0.01 ≤ *P* value < 0.05 as ‘c’. This notation aids in quickly identifying the strength of the statistical findings reported in the study results.

## Result

The study analyzed 30,855 discharges related to Diseases and Disorders of the Circulatory System (DDCS). Heart Failure and Shock (HFS) was the most prevalent, representing 47.2% of discharges. This was followed by Cardiac Arrythmia and Conduction Disorders (CACD) and Acute Myocardial Infarction (AMI) each accounting for 12.3% and 9.9%, respectively. Hypertension (HTN) and Chest Pain (CP) were also significant, noted in 6.4% and 6.2% of cases. In this study, the analysis focused on DDCS with frequencies greater than 5%, specifically HFS, CACD, Circulatory Disorders Except for AMI with Cardiac Catheterization (CDEAMI), AMI, HTN, and CP.

Less common conditions included Syncope and Collapse at 4.7%, Peripheral Vascular Disorders at 4.4%, and Atherosclerosis at 1.8%. Rare disorders such as Unexplained Cardiac Arrest, Angina Pectoris, Cardiac Congenital and Valvular Disorders, Acute and Subacute Endocarditis, and Deep Vein Thrombophlebitis collectively made up less than 1% of the hospital discharges each. Other Circulatory System Diagnoses accounted for 6.0% of the discharges (Table 1).

**Table 1:** Summary of hospital discharges with diseases & disorders of the circulatory system

Diseases & Disorders	*n* (%)
Total Discharge	30,855
Heart Failure and Shock (HFS)	13,112 (47.2%)
Cardiac Arrhythmia and Conduction Disorders (CACD)	3,427 (12.3%)
Circulatory Disorders Except for AMI with Cardiac Catheterization (CDEAMI)	3,057 (9.9%)
Acute Myocardial Infarction (AMI)	2,737 (9.9%)
Hypertension (HTN)	1,782 (6.4%)
Chest Pain (CP)	1,717 (6.2%)
Syncope and Collapse (SC)	1,307 (4.7%)
Peripheral Vascular Disorders (PVD)	1,208 (4.4%)
Atherosclerosis (Ath)	504 (1.8%)
Unexplained Cardiac Arrest (CAU)	114 (0.4%)
Angina Pectoris (AP)	112 (0.4%)
Cardiac Congenital and Valvular Disorders (CCVD)	61 (0.2%)
Acute and Subacute Endocarditis (ASE)	33 (0.1%)
Deep Vein Thrombophlebitis (DVT)	12 (0.04%)
Other Circulatory System Diagnoses (OCSD)	1,672 (6.0%)

**Fig. 1:**
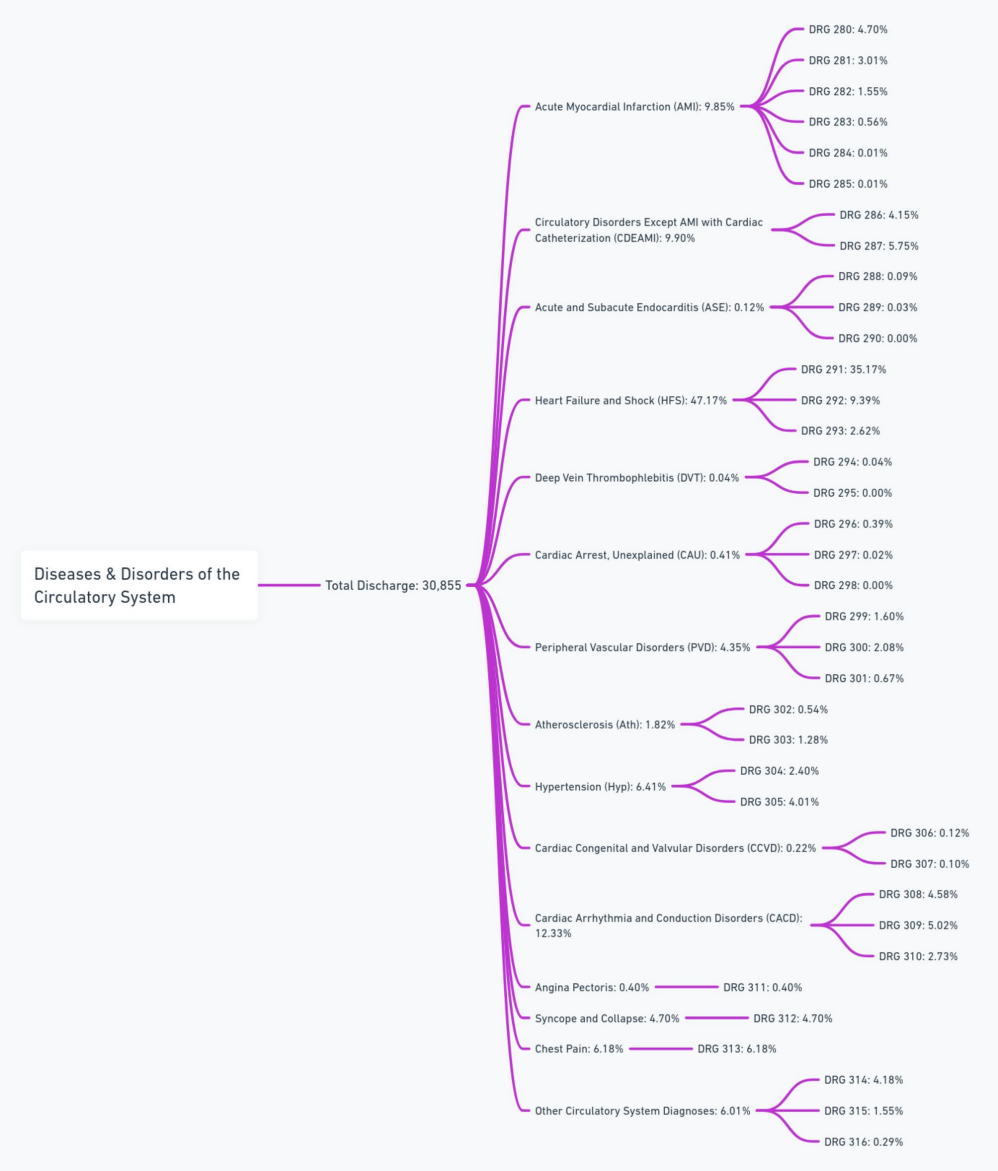
Distribution of Hospital Discharges by Cardiovascular DRGs

Figure 1 illustrates the distribution of patient’s hospital discharges by DRGs for various CVDs. DRGs are categorized based on the severity and type of conditions as outlined by the CMS. For detailed DRG coding and classification, refer to the CMS ICD-10 Version 37 Full Code List, available at CMS (Fig. 1).

Demographic analysis revealed distinct patterns across cardiovascular conditions. Hypertension affected a higher proportion of females (54.7% of HTN patients) compared to males. Patients with acute myocardial infarction had the highest mean age (65.2 years) among all cardiovascular conditions studied. Within our study population, African Americans represented the overwhelming majority across all cardiovascular disease categories, particularly in hypertension cases (98.9%), reflecting the demographic composition of our study cohort.

Most of the cases of HFS (92.4%) were admitted through Emergency. While the highest proportions (78.6%, and 75.6%) of hospital discharge to home or self-care is for patients admitted due to CP and HTN respectively.

In terms of co-morbidities, around 61% of HFS patients have three or more co-morbidities with more than half of HFS patients have renal failure. The hospital length of stay varies by CVD condition with the longest stays being noted in CDEAMI cases at an average of 5.4 days. Geographically, Norfolk had the highest proportion of patients across most conditions, notably HFS (23.4%) (Table 2).

**Table 2:** Demographic, administrative, clinical, and comorbidity characteristics of patients with circulatory system disorders

	HFS	CACD	CDEAMI	AMI	HTN	CP
Female, %	49.9	50.4	49.5	47.2	54.7	52.8
Age, Mean ± SD	64.8 ± 13.7	64.0 ± 14.3	59.2 ± 12.7	65.2 ± 13.5	54.8 ± 14.8	61.1 ± 13.8
African American, %	98.7	97.8	98.7	98.0	98.9	98.4
Medicare, %	63.9	57.9	45.9	58.7	37.5	57.8
Medicaid, %	10.0	8.8	11.4	7.9	14.5	8.8
Self-Pay, %	5.8	6.1	8.9	7.5	16.6	8.2
Other Insurance, %	19.9	27.3	33.8	25.9	31.4	27.1
Emergency Admit, %	92.4	90.3	83.0	88.4	91.1	92.7
Urgent Admit, %	6.4	5.4	9.4	9.2	7.2	6.1
Elective Admit, %	1.1	3.91	7.6	2.2	1.7	1.1
Non-HCF Admit Source, %	71.5	68.2	58.8	72.2	78.8	80.3
COA Admit Source, %	24.1	26.4	31.8	18.6	18.9	16.4
HSC Discharge Status, %	45.6	63.8	62.9	47.5	75.6	78.6
OHC Discharge Status, %	35.6	21.0	21.0	20.9	13.9	13.1
With Primary Procedure %	78.1	74.0	100	83.4	68.3	76.1
With Complication %	2.1	2.0	5.0	3.8	1.1	1.5
0 Comorbidity, %	3.7	10.7	10.7	8.4	7.6	9.3
1 Comorbidity, %	12.3	22.5	23.9	18.7	16.1	21.3
2 Comorbidity, %	22.2	25.0	26.2	23.5	21.1	27.0
3 Comorbidity, %	25.9	20.2	20.3	23.0	19.9	20.4
4 Comorbidity, %	20.0	12.6	12.8	16.4	15.4	12.9
5 + Comorbidity, %	15.9	9.0	6.1	10.0	19.9	9.1
DCC, %	46.7	24.3	31.9	37.9	31.3	32.9
Renal Failure, %	58.1	27.7	28.8	41.0	42.7	31.1
Obesity, %	37.1	32.4	36.2	24.6	31.3	29.4
FED, %	34.6	29.4	27.6	31.4	32.4	19.1
Deficiency Anemias, %	37.4	22.7	22.3	28.2	26.6	25.7
Los, Mean ± SD	5.1 ± 4	3.5 ± 3	5.4 ± 5	4.6 ± 4	3.3 ± 3	2.3 ± 1.92
Pre-Op LOS, Mean ± SD	1.0 ± 2	0.9 ± 2	2.6 ± 3	1.5 ± 2	0.8 ± 2	0.6 ± 1.14
Post-Op Los, Mean ± SD	4.4 ± 4	2.9 ± 3	2.9 ± 4	3.4 ± 4	2.8 ± 3	1.9 ± 2
City of Norfolk, %	23.4	19.5	16.8	18.7	21.8	28.8
City of Newport News, %	13.1	14.2	13.2	13.5	14.4	11.4
City of Virginia Beach, %	13.4	15.8	16.4	12.4	14.7	15.4
City of Chesapeake, %	11.7	13.0	11.1	15.7	16.4	10.5
City of Hampton, %	11.6	12.8	14.0	14.0	9.2	10.4

*Categorical variables are presented as n(%). Continuous variables are presented as mean ± SD. HFS: Heart Failure and Shock; CACD: Cardiac Arrhythmia and Conduction Disorders; CDEAMI: Circulatory Disorders Except for AMI with Cardiac Catheterization; AMI: Acute Myocardial Infarction; HTN: Hypertension; CP: Chest Pain; COA: Clinic/Physician’s Office Admission; HSC: Home or Self-Care; OHC: Organized Home Care Discharge; DCC: Diabetes with Chronic Complications; FED: Fluid and Electrolyte Disorders; LOS: Length of Stay. All regions listed are metropolitan cities within the Southeastern Virginia urban area. These cities comprise the core of the Hampton Roads metropolitan area, which has a population of over 1.7 million residents

Substantial gender differences were noted, with females showing significantly higher proportions in AMI and HTN − 5.7% (b) and 9.3% (a) respectively. Age disparities were marked, with younger patients (< 65) exhibiting significant differences in conditions such as HTN, where the disparity reached 50.3% (a). Mean age by gender differences were significant across all conditions, with HTN showing the highest difference at 4.8% (a).

Medicare coverage differences were particularly stark in HFS at 27.8% (a). The analysis also highlighted that the presence of primary procedures (CPT) and complications contributed to significant variances across all conditions, consistently high above 90%. Admission type variations revealed significant differences, especially between emergency and elective admissions, most notably in HFS at 91.4% (a).

Significant differences based on the care admission sources observed, with non-healthcare facility admissions incurring notably higher charges in conditions such as HTN at 57.6% (a). Gender-related differences in the average length of hospital stay were significant for HFS and AMI, with females generally experiencing on average longer hospital stays. No significant differences were observed in pre-operative lengths of stay, while post-operative lengths of stay showed significant disparities in HFS, CACD, and HTN (Table 3).

**Table 3:** Comparative analysis of demographic, clinical, and administrative differences in circulatory system disorders

	Group Difference							
	Comparison (A vs. B or A-B)	HFS	CACD	CDEAMI	AMI	HTN	CP	
Gender, %	F vs. M	-0.2	0.8	-0.9	-5.7^b^	9.3 ^a^	5.5^c^	
Age, %, y	< 65 vs. >=65	-4.9^a^	-4.9^b^	30.7 ^a^	-7.1^b^	50.3 ^a^	16^a^	
Age by Gender, Mean	F vs. M	3.5^a^	4.4 ^a^	2.3 ^a^	4.3 ^a^	4.8 ^a^	2.2^a^	
Medicare, %	Yes vs. No	27.8^a^	15.8 ^a^	-8.14^a^	17.4 ^a^	24.9 ^a^	-15.7 ^a^	
CPT, %	Yes vs. No	56.1 ^a^	48^a^	100	66.8 ^a^	36.6 ^a^	52.2 ^a^	
Complication, %	Yes vs. No	-95.9 ^a^	-96 ^a^	-90.1 ^a^	-92.3 ^a^	-97.8 ^a^	-97 ^a^	
Admit Type, %	Em vs. Ur	86 ^a^	84.9 ^a^	73.6 ^a^	79.2 ^a^	84.0 ^a^	86.5 ^a^	
	Em vs. El	91.4 ^a^	86.4 ^a^	75.3 ^a^	86.2 ^a^	89.5 ^a^	91.6 ^a^	
	Ur vs. El	5.4 ^a^	1.5^b^	1.8^c^	7^a^	5.5	5^a^	
Admit Source, %	NHCFA vs. Other	43 ^a^	36.5 ^a^	17.6 ^a^	44.4^b^	57.6 ^a^	60.6 ^a^	
LOS, Mean	F vs. M	0.5 ^a^	0.05	0.1	0.3^b^	0.3^b^	0.1^b^	
Pre-Operative LOS, Mean	F vs. M	0.1	-0.03	0.01	0.2	0.1	-0.03	
Post-Operative LOS, Mean	F vs. M	0.4 ^a^	0.1^c^	0.1	0.1	0.2^c^	0.1^c^	

*Group differences for categorical variables were assessed using Chi-square tests or Fisher’s exact tests. For continuous variables, t-tests were used for normally distributed data and Wilcoxon rank-sum tests for non-normally distributed data. Multiple comparisons were controlled using the Benjamini-Hochberg method. Sig: Significant level of the *P* value; CPT: Current Procedural Terminology (indicating primary procedure); EM: Emergency; Ur: Urgent; Elective: El; NHCFA: Non-Health Care Facility Admission; LOS: Length of Stay; a: *P* value < 0.0001; b: A 0.0001 ≤ *P* value < 0.01; c: 0.01 ≤ *P* value < 0.05

Significant variations in total charges were observed across various demographic and clinical groups for patients with and without primary procedures. Notably, gender differences emerged as a significant factor in healthcare costs, with females incurring 7.1% (a) higher charges than males when a CPT was involved and 8.5% (a) higher without CPT in HFS. Similarly, age impacted charges, particularly in CACD, where younger patients (< 65) faced 8.5% (a) higher charges with CPT and a lesser but still notable increase of 2.6% without CPT.

In CDEAMI, where CPT is always involved due to the nature of the condition, younger patients experienced a 5.2% (c) charge increase. Medicare’s influence was also profound, associated with higher charges in CACD, showing a 9.7% (a) increase with CPT and 4.6% without, and in CDEAMI with a 7.9% (a) increase with CPT. The impact of complications was significantly noted across all conditions, particularly in HTN, with a 38.6% (c) increase with CPT and an even more substantial 46.3% increase without CPT. In HFS, complications led to 35.4% (a) higher charges with CPT and 34.5% (c) higher without (Table 4).

**Table 4:** Total charge group differences (%)

	Difference (%) with CPT^Sig^| Difference (%) without CPT^Sig^										
	Comparison (A vs. B or A-B)	HFS	CACD		CDEAMI^#^		AMI		HTN	CP	
Gender	F vs. M	7.1^a^|8.5^a^		2.32|6		2.93| -		0.83|1.8	1.8| 4.4	8.8^b^|13.9^c^	
Age	< 65 vs. >=65	4^b^| 2.8^b^		8.5^a^|2.6		5.2^c^| -		5.5^c^|1.9	0.9|2	4.0|9.2	
Medicare	With vs. Other	0.2|7.4^b^		9.7^a^|4.6		7.9^a^| -		4.9|5.5	2.6|5.2	4.3|9.6	
Complication	With vs. Without	35.4^a^|34.5^c^		30.7^a^|18.3		29.2^a^| -		25.6^a^|31^a^	38.6^c^|46.3	9.7|15.7	
Admission Type	EM vs. Other	7.7^c^|4.5		28.9 ^a^|10^c^		12.6 ^a^| -		9.6|9.2	11.5|2.6	22.3^c^|6.3	
Admission Source	NHCFA vs. Other	3|12.7		5|23.5^c^		11.3 ^a^| -		13.8^a^|23.6	3.1|26.3	2.4|24.5	
	HSD vs. NHSD	28.5^a^|25.6^a^		34.7^a^|29.3^a^		28 ^a^| -		19.1^a^|7.5^c^	13.7^a^|17.7^a^	14.5^a^|3.6	
Number of Comorbidities	0 vs. 1	14.4^a^|4.1		13.8^b^|0.5		8.2^b^| -		7.2^b^|12.9	18.6^c^|2.8	4.0|2.3	
	1 vs. 2	9.6^a^|6.4^c^		8.2^b^|15.5^b^		11.4^a^| -		3.6|7.6	7.0|2.3	6.3|14.1	
	2 vs. 3	7.3^a^|11^b^		7.2^b^|10.8		6.7^b^| -		9.6^b^|10.3	15.4^b^|3.8	0.3|10.1	
	3 vs. 4	9.9^a^|4.5		12.3^b^|6.8		17.6 ^a^| -		1.4|6.5	10.9|0.2	12.1^b^|6.1	
	4 vs. 5+	6.5^b^|2.3		5.1|17.8		1.4|-		7|14.2	0.1|18.7^c^	7.0|6.9	

*Differences in total charges were analyzed using t-tests for normally distributed data and Wilcoxon rank-sum tests for non-normally distributed data. Data were transformed as needed to achieve normality. Multiple group comparisons were conducted using ANOVA or Kruskal-Wallis tests. Sig: Significant level of the *P* value; CPT: Procedure; #: CDEAMI always involves a catheterization procedure, hence there are no records available for this condition without a primary procedure; EM: Emergency; Admit-S: Admit Source; Admit-T: Admit Type; HSD: Home Selfcare Discharge; NHSD: non-Home Selfcare Discharge; Comorbidity: Number of comorbidity; ^a^: *P* value < 0.0001; ^b^: 0.0001 ≤ *P* value < 0.01; ^c^: 0.01 ≤ *P* value < 0.05

Differences in admission type further influenced the total charges, particularly in CACD, where emergency admissions resulted in 28.9% (a) higher charges with CPT and 10.0% (c) higher without. The source of admission also marked significant differences, especially in CDEAMI, with an 11.3% (a) increase with CPT and in AMI, with 13.8% (a) with CPT and 23.6% without. HSD was consistently associated with significantly higher charges across all conditions, particularly noted in CACD, which show 34.7% (a) higher charges with and without CPT. Furthermore, the number of comorbidities played a crucial role in total charges, with HFS showing a 14.4% (a) increase from 0 to 1 comorbidity with CPT and a 6.4% (c) increase from 1 to 2 comorbidities without CPT. Notably, a 9.9% (a) increase from 3 to 4 comorbidities with CPT and an 11.0% (b) increase from 2 to 3 comorbidities without CPT highlighted the escalating cost impact related to the rising number of comorbidities (Table 4).

Table 5 reveals that models incorporating CPT generally offer better predictive accuracy, with R² values ranging from 0.80 to 0.87 compared to 0.64 to 0.74 for models without CPT. This underscores the value of including procedural data in predicting healthcare costs. Age inversely affects charges in AMI, where older patients typically incur lower charges, evidenced by a decrease of -0.333% (a) with CPT and − 0.430% (c) without CPT. This suggests younger patients may undergo more intensive or costly treatments. Additionally, the year of discharge consistently correlates with an increase in charges across all conditions, notably in AMI at 0.816% (a) with CPT and 1.085% (a) without, and in CP at 0.845% (a) with CPT and 0.913% (a) without, indicating a trend of rising healthcare costs over time.

**Table 5:** Factors associated with total charge

	With CPT^Sig^| without CPT^Sig^					
	HFS	CACD	CDEAMI^#^	AMI	HTN	CP
MP (R²)	0.84| 0.64	0.84| 0.64	0.87| -	0.80| 0.70	0.86| 0.73	0.87| 0.74
Standardized Regression Coefficient						
Age	-0.111^a^|-0.085	-0.180^b^|0.045	-0.123^b^| -	-0.333^a^|-0.430^c^	-0.106^c^|-0.062	-0.128^c^|-0.084
Year of Discharge	0.189^b^|0.841^a^	0.246^b^|0.721^a^	0.430^a^| -	0.816^a^|1.085^a^	0.298^c^|1.222^a^	0.845^a^|0.913^a^
Number of comorbidities	0.024|0.134^a^	0.018|0.147^b^	0.016| -	-0.080^b^|0.214^b^	0.053|0.131^c^	-0.004|0.095
Hospital Length of Stay	1.341| -	0.110| -	0.747^a^| -	2.026| -	0.833| -	0.052| -
PRLOS	-0.203| -	0.244| -	-0.117^a^| -	-0.601| -	-0.102| -	0.174| -
PSLOS	-0.465| -	0.554| -	0| -	-1.021| -	-0.143| -	0.435| -
Sex="M”	-0.004|-0.021	0.011|0.047	0.009| -	0.017|-0.025	0.020|-0.011	-0.011|-0.045
Complication	0.028|0.027^c^	-0.005|0.029	0.059^a^| -	0.032^b^|0.061^c^	-0.008|-0.020	0.024^c^|-0.002
Emergency	0.046|-0.014	0.071|0.036	0.012| -	-0.167^b^|0.092	0.083|-0.367^b^	-0.301^c^|0.08
Trauma	-0.001|0.015	0.018^c^|0.050^c^	-0.0008| -	-0.008|0.079^b^	0.015| -	0.010|0.048
Urgent	-0.002|-0.034	-0.027^c^|-0.033	-0.017| -	-0.069^b^|0.039	0.021| -0.16^b^	-0.073^b^|0.005
Medicare	0.0298^b^|-0.014	0.025|-0.141^c^	0.034^c^| -	0.014|-0.128	0.012| -0.012	0.056|-0.043
Other Insurance	0.0179^b^|-0.036	0.004|-0.089^c^	0.001| -	-0.013|-0.107^c^	-0.003| -0.049	0.025|-0.078
Self-Pay	0.0004|-0.040^b^	-0.007|-0.037	-0.006| -	-0.016|-0.084^c^	0.008| -0.053	0.009|-0.022
(DM = 1)	-0.008|-0.022	-0.011|0.009	-0.004| -	0.012|0.014	-0.037|0.004	0.007|-0.012
(RENLFAIL = 1)	-0.029^a^|-0.024	-0.009|-0.020	-0.022^c^| -	-0.023|0.048	-0.008|0.044	-0.009|-0.078^c^
(OBESE = 1)	-0.011^c^|0.007	-0.021|-0.025	-0.027^b^| -	-0.003|-0.070^c^	0.034|0.048	-0.022|-0.030
(LYTES = 1)	0.042^a^|0.037^c^	0.046^a^|0.042	0.007| -	0.051^a^|0.087^c^	-0.025^c^|0.007	0.008|0.0197
(ANEMDEF = 1)	-0.019b|-0.008	-0.021^c^|0.005	-0.013| -	-0.032|-0.026	-0.106|-0.064^c^	-0.038^c^|0.074^c^

* CPT: Current Procedural Terminology (indicating primary procedure); Sig: Significance Level; MP: Model Performance (R²); DM: Diabetes Mellitus; RENLFAIL: Renal Failure; OBESE: Obesity; LYTES: Fluid and Electrolyte Disorders; ANEMDEF: Deficiency Anemias; PRLOS: Pre-operative Length of Stay; PSLOS: Post-operative Length of Stay. Multiple regression models were used to identify factors associated with total charges. Standardized regression coefficients are reported. All variables presented in this table remained after the Variance Inflation Factor (VIF) analysis, with variables having VIF > 10 removed from the models. The highest VIF value among the retained variables was 3.6 (for number of comorbidities), indicating no substantial concerns regarding multicollinearity in the final models. Standardized regression coefficients are reported. Significance levels: ^a^: *P* value ≤ 0.0001; ^b^: 0.0001 ≤ *P* value < 0.01; ^c^: 0.01 ≤ *P* value < 0.05

Comorbidities significantly impact charges, especially in HFS, with an increase of 0.134% (a) and in CACD at 0.147% (b) without CPT. This association suggests higher charges accompany greater health burdens. Emergency admissions displayed lower charges in AMI at -0.167% (b) with CPT, highlighting how different admission types can influence healthcare costs. Conditions such as renal failure and electrolyte disorders are significant cost drivers, with renal failure showing a negative association in HFS at -0.029% (a) with CPT and electrolyte disorders indicating positive associations in HFS at 0.042% (a) with CPT and 0.037% (c) without. These findings emphasize the complex interplay of clinical and administrative characteristics in determining healthcare costs and highlight the substantial financial implications of specific medical conditions and treatment modalities (Table 5).

## Discussion

Our study highlighted significant gender differences in CVDs, with females showing higher incidences of CACD, and HTN, and CP. These findings are supported by literature suggesting gender-specific variations in CVD prevalence, influenced by biological and social factors [24, 25]. Specifically, the higher prevalence of HTN in females aligns with recent studies on gender differences in cardiovascular health [26]. However, our finding of higher AMI rates in females contrasts with several previous studies [27–30], that typically report higher prevalence in males. This unexpected finding suggests potential regional or demographic specificities in our Southeastern Virginia population. Our female patients with AMI were significantly older than males (mean age difference of 4.3 years, *p* < 0.0001), which could partially explain this difference. Additionally, the high prevalence of comorbidities known to disproportionately affect African American women in our study cohort—including diabetes with chronic complications (37.9% of AMI patients), renal failure (41.0%), and obesity (24.6%)—likely contributes to this gender disparity. Our observations of longer post-operative lengths of stay for females and the high rate of emergency admissions (88.4% of AMI patients) further suggest that complex interactions between gender, access to care, and presentation patterns may influence AMI outcomes in this predominantly African American population. This gender disparity in CVD prevalence may reflect a complex interplay of biological factors and social determinants of health specific to the Southeastern Virginia region [17, 25, 31]. Compared to previous studies [27–30], the contrast in AMI rates underscores the importance of considering local population characteristics when developing cardiovascular health strategies. These findings highlight the need for gender-specific approaches in CVD prevention, diagnosis, and treatment, particularly in regions with demographic profiles similar to Southeastern Virginia [24, 28, 32]. The disparities were also prominent, with younger patients (< 65) displaying higher rates of HTN and CP, aligning with established research that identifies age as a critical factor in CVDs risk and management [33, 34]. The higher prevalence of HTN in younger patients is particularly concerning, as it suggests a potential shift in the age distribution of CVD risk factors. This trend may be attributed to changing lifestyle factors, increased stress, or earlier onset of obesity in younger populations [35–38].

Significant differences in insurance coverage were observed, with Medicare predominantly used by HFS patients, contrasting with a higher reliance on self-pay among HTN patients. This observation is consistent with studies indicating that insurance type significantly affects access to care and outcomes in CVDs treatment [39–41]. The higher rate of self-pay among HTN patients is particularly troubling, as it may lead to delayed diagnosis and treatment, potentially resulting in more severe cardiovascular outcomes as HTN itself is an underlying risk factor of other chronic conditions such as heart failure, renal failure, and stroke [42–44]. This finding highlights the need for policy interventions to improve access to preventive care and chronic disease management for uninsured and underinsured populations. The presence of multiple comorbidities was a significant predictor of worse CVDs outcomes, especially in HFS where a majority of patients had three or more comorbid conditions. This underscores the importance of managing comorbid conditions to improve health outcomes, as multiple comorbidities complicate treatment approaches and impact patient prognosis significantly [45–47].

Our findings on the impact of comorbidities are consistent with recent studies emphasizing the need for integrated care models in managing patients with multiple chronic conditions [2, 48, 49]. The high prevalence of renal failure among HFS patients (58.1%) reflects the significant burden of this comorbidity and its economic implications for healthcare systems. This finding aligns with national data showing the substantial impact of renal dysfunction on healthcare utilization and costs in cardiovascular patients [50, 51]. From an economic and health disparity perspective, addressing renal complications in cardiovascular patients could potentially reduce the financial burden on both patients and healthcare systems while improving outcomes in minority populations where these comorbidities are disproportionately prevalent.

Findings on hospitalization patterns highlighted the predominance of emergency admissions, particularly in HFS, and higher discharge rates to home or self-care in HTN and CP cases. These patterns reflect the urgency and severity of CVDs presentations, emphasizing the need for efficient emergency care and robust post-discharge support to reduce readmissions [52–54]. The high rate of emergency admissions, especially for HFS, suggests potential gaps in outpatient management and preventive care. Implementing more robust community-based interventions and improving access to primary care could help reduce the burden on emergency services and improve overall patient outcomes [55, 56].

Our findings revealed consistent gender disparities in healthcare costs, with females generally incurring higher charges than males across various cardiovascular conditions. While our database does not provide granular details on specific treatments or insurance coverage limitations that might explain these differences, several factors may contribute to this pattern. First, female patients in our study were significantly older than males across all conditions (with mean age differences of 2.2–4.8 years), potentially leading to more complex presentations requiring more intensive interventions. Second, we observed longer post-operative lengths of stay for females in several conditions (HFS, CACD, HTN, and CP), directly increasing hospitalization costs. Third, the literature documents gender-specific differences in CVD presentation, with females often showing more atypical symptoms that may lead to more extensive diagnostic testing or differential treatment approaches [57]. Additionally, physiological differences between males and females, such as smaller coronary arteries in females, may necessitate specialized equipment or techniques during procedures, potentially affecting costs. As noted by Mosca et al. (2011), these biological and presentation differences can translate into disparities in healthcare utilization and associated costs [58]. Future research incorporating more detailed clinical data, including specific treatments, procedures, and insurance coverage details, would be valuable to further elucidate the mechanisms underlying these gender-based cost disparities and to develop targeted interventions to address them.

The study also noted geographic variations in CVDs incidence and management, with urban centers like Norfolk exhibiting higher patient proportions. This suggests that local demographic, socioeconomic, and healthcare infrastructure factors significantly influence CVDs outcomes [59]. These findings align with recent studies highlighting the importance of place-based interventions in addressing cardiovascular health disparities [12]. The concentration of cases in urban areas like Norfolk may reflect a combination of factors including population density, environmental stressors, and potentially unequal distribution of healthcare resources [60].

Furthermore, our cost analysis indicated substantial economic impacts related to various patient and clinical factors, with the presence of complications and the number of comorbidities significantly driving up healthcare costs, highlighting the financial strain associated with more complex CVDs cases. The strong association between comorbidities and increased costs underscores the potential economic benefits of integrated care models and preventive strategies that address multiple chronic conditions simultaneously. Policy makers and healthcare systems should consider these findings when allocating resources and designing interventions to manage CVDs more cost-effectively.

This study relies on administrative hospital discharge data from the VHI database, which presents several inherent limitations. First, these data were collected primarily for billing and administrative purposes rather than research, potentially affecting the depth and focus of clinical information available. Although VHI implements rigorous editing procedures at the record level to maintain data integrity, important constraints remain. Coding practices may vary substantially across healthcare facilities, leading to inconsistent documentation of diagnoses and procedures. The retrospective nature of the data limits our ability to establish causality, and the lack of granular clinical details beyond recorded diagnoses and procedures restricts more nuanced clinical interpretations. Furthermore, certain conditions might be misclassified or underreported in administrative data, particularly those that are not primary drivers of reimbursement. While our study identified complications as a significant cost driver, the administrative nature of the VHI database limited our ability to analyze specific complication types (bleeding, vascular injury, contrast-induced nephropathy, etc.). Future studies using clinical registries or detailed electronic health records would provide better insights into specific procedural complications and their economic impacts across demographic groups. Despite the inherent limitations of administrative data, this study provides valuable insights into CVD patterns among minority populations in Southeastern Virginia. The large sample size (30,855 discharges) enables robust statistical analyses that reveal significant patterns in healthcare utilization, costs, and outcomes. While administrative data lacks some clinical granularity, it captures system-level trends and disparities that are critical for healthcare planning and policy development. Our findings on gender differences, insurance coverage patterns, and comorbidity impacts offer actionable information for developing targeted interventions, even with the acknowledged limitations of retrospective administrative data. Our study’s categorization of cardiovascular conditions follows standardized DRGs and ICD coding practices, which has inherent limitations. Categories like ‘Heart Failure and Shock’ and ‘Chest Pain’ represent broad administrative groupings rather than clinically validated diagnoses. Without access to detailed clinical data such as catheterization findings or biomarker results, we cannot distinguish between specific subtypes of conditions or verify diagnostic accuracy beyond the coded information available in administrative records. While the RATs data significantly enhanced our ability to track episodes of care, it too has limitations. The matching algorithm depends on consistent recording of patient identifiers across encounters, which may not be uniform across all facilities or patient encounters. Additionally, since VHI does not require Social Security Numbers for patients three years or younger, the RATs information for this demographic group is incomplete. Though this particular limitation minimally impacts our CVD study, which focused primarily on adult populations, it represents an important constraint of the data source that should be acknowledged for methodological transparency. Finally, while our study intentionally focused on AA&H populations in Southeastern Virginia without comparison to other racial/ethnic groups, national data provides context for our findings. According to the American Heart Association’s 2023 report, CVD mortality rates for African Americans exceed those of non-Hispanic whites by approximately 30% nationwide [1]. Our findings regarding high emergency admission rates (> 90% for several conditions) and substantial comorbidity burdens (> 60% of HFS patients having three or more comorbidities) align with national patterns of cardiovascular disparities in minority populations [61, 62]. Future research should directly compare outcomes between racial/ethnic groups within Southeastern Virginia to better quantify local disparities and evaluate the effectiveness of targeted interventions.

## Conclusion

This comprehensive analysis of CVDs among a diverse population in Southeastern Virginia highlights significant disparities in disease prevalence, healthcare utilization, and outcomes. Key findings revealed that demographic factors such as gender and age substantially impact disease prevalence and healthcare costs, with females having higher incidences of chronic conditions like HTN and males being more affected by conditions like AMI. The study identified insurance type as a critical factor influencing healthcare access and outcomes, with Medicare beneficiaries showing different utilization patterns compared to other groups. Comorbidities significantly increased hospitalization costs and care complexity, with conditions like renal failure being particularly prevalent.

Our cost analysis revealed significant disparities influenced by gender, admission source, and admission type, underscoring the economic implications of these demographic and clinical factors. These findings contribute valuable insights into CVD management within minority populations and support the development of targeted interventions to reduce disease burden, improve outcomes, and mitigate economic strain on healthcare systems serving diverse communities.

### Implications of findings

The results of this study underscore the need for healthcare policy improvements that enhance access to preventive and ongoing care for underinsured groups, particularly in managing CVDs. Given the significant impacts of gender and age on CVD outcomes, tailored clinical approaches and targeted interventions could be more effective, such as lifestyle modification programs for younger patients and enhanced screening for older individuals. The high prevalence of comorbidities like renal failure and diabetes among CVD patients calls for integrated care models that coordinate across specialties to improve overall patient outcomes.

Additionally, the substantial rates of emergency admissions and economic implications highlighted by this study underline the importance of optimizing emergency care protocols and strengthening post-discharge support to reduce readmissions and associated costs. Geographic variations in CVD incidence suggest that localized health interventions could significantly reduce disease burden in high-prevalence areas. Lastly, the financial implications associated with demographic and clinical factors warrant further research into cost-effective healthcare delivery models, aiming to balance clinical effectiveness and cost-efficiency to enhance equitable and sustainable management of CVDs.

## Data Availability

The datasets used during the current study are derived from the Virginia Health Information (VHI) Patient Level Database and the Readmissions and Transfers Supplemental Data Set (RATs), which are licensed inpatient hospital discharge data files containing all submitted, processed, and verified discharges in the Commonwealth of Virginia. The data were accessed through the M. Foscue Brock Institute for Community and Global Health at Macon & Joan Brock Virginia Health Sciences at Old Dominion University and the Research and Infrastructure Service Enterprise at Macon & Joan Brock Virginia Health Sciences at Old Dominion University. The data are not publicly available due to VHI licensing agreements and privacy restrictions but may be available from the corresponding author upon reasonable request and subject to approval by VHI and the M. Foscue Brock Institute for Community and Global Health at Macon & Joan Brock Virginia Health Sciences at Old Dominion University. Additional information about the data extraction methodology and the process used in this project to link the Patient Level Database to the RATs can be provided by contacting Dr. Ismail El Moudden at elmoudi@odu.edu. Researchers interested in accessing similar data should contact VHI directly to complete the appropriate license agreement and pay applicable fees. The data are held under the terms stipulated by the VHI licensing agreement, which prohibits public sharing of the data to protect patient confidentiality and comply with legal restrictions. Information about obtaining VHI data can be found at www.vhi.org/pld.

## Abbreviations

CVD: Cardiovascular Disease
VHI: Virginia Health Information
AA&H: African American and Hispanic
HFS: Heart Failure and Shock
CACD: Cardiac Arrhythmia and Conduction Disorders
HTN: Hypertension
CP: Chest Pain
AMI: Acute Myocardial Infarction
RATs: Readmissions and Transfers Supplemental Data Set
MDC: Major Diagnostic Category
DRG: Diagnosis Related Group
CMS: Centers for Medicare and Medicare Services
IRB: Institutional Review Board
RISE: Research and Infrastructure Service Enterprise
IQR: Interquartile Range
VIF: Variance Inflation Factor
DDCS: Diseases and Disorders of the Circulatory System
CDEAMI: Circulatory Disorders Except for AMI with Cardiac Catheterization
CPT: Primary procedure
